# Synchronous Defect and Interface Engineering of NiMoO_4_ Nanowire Arrays for High-Performance Supercapacitors

**DOI:** 10.3390/nano12071094

**Published:** 2022-03-26

**Authors:** Pengcheng Wang, Xinying Ding, Rongjie Zhe, Ting Zhu, Chen Qing, Yingkai Liu, Hong-En Wang

**Affiliations:** 1 Yunnan Key Laboratory of Optoelectronic Information Technology, College of Physics and Electronics Information, Yunnan Normal University, Kunming 650500, China; pcwang0312@163.com (P.W.); xinyingding1998@163.com (X.D.); rongjiezhe@163.com (R.Z.); zhut0002@ynnu.edu.cn (T.Z.); ykliu@ynnu.edu.cn (Y.L.); 2 Key Laboratory of Advanced Technique & Preparation for Renewable Energy Materials, Ministry of Education, Yunnan Normal University, Kunming 650500, China

**Keywords:** NiMoO_4_ nanowire arrays, oxygen vacancies, core-shell electrode structure, asymmetric supercapacitors

## Abstract

Developing high-performance electrode materials is in high demand for the development of supercapacitors. Herein, defect and interface engineering has been simultaneously realized in NiMoO_4_ nanowire arrays (NWAs) using a simple sucrose coating followed by an annealing process. The resultant hierarchical oxygen-deficient NiMoO_4_@C NWAs (denoted as “NiMoO_4−*x*_@C”) are grown directly on conductive ferronickel foam substrates. This composite affords direct electrical contact with the substrates and directional electron transport, as well as short ionic diffusion pathways. Furthermore, the coating of the amorphous carbon shell and the introduction of oxygen vacancies effectively enhance the electrical conductivity of NiMoO_4_. In addition, the coated carbon layer improves the structural stability of the NiMoO_4_ in the whole charging and discharging process, significantly enhancing the cycling stability of the electrode. Consequently, the NiMoO_4−*x*_@C electrode delivers a high areal capacitance of 2.24 F cm^−2^ (1720 F g^−1^) at a current density of 1 mA cm^−2^ and superior cycling stability of 84.5% retention after 6000 cycles at 20 mA cm^−2^. Furthermore, an asymmetric super-capacitor device (ASC) has been constructed with NiMoO_4−*x*_@C as the positive electrode and activated carbon (AC) as the negative electrode. The as-assembled ASC device shows excellent electrochemical performance with a high energy density of 51.6 W h kg^−1^ at a power density of 203.95 W kg^−1^. Moreover, the NiMoO_4_//AC ASC device manifests remarkable cyclability with 84.5% of capacitance retention over 6000 cycles. The results demonstrate that the NiMoO_4−*x*_@C composite is a promising material for electrochemical energy storage. This work can give new insights on the design and development of novel functional electrode materials via defect and interface engineering through simple yet effective chemical routes.

## 1. Introduction

Several alternative energy technologies have been under development globally in a great effort to mitigate the energy and environmental challenges faced and in accordance with the current “carbon neutral” policies. Supercapacitors (SCs), also known as electrochemical capacitors (ECs), have been considered as one of the most promising energy storage devices due to their unique characteristics of high power density (>10 kW/kg), fast charging and discharging capability (within a few seconds), long lifespan (over 100,000 cycles), and good operational safety. SCs have been widely applied in some important fields, including smart electric grids, memory back-ups, (hybrid) electric vehicles, and aerospace crafts. Although SCs have the advantages of high power density and very long calendar lives, their further application is still hindered by their limited energy density. Therefore, it is crucial to develop advanced high-performance SCs with higher energy densities without severely compromising the power density and cyclability.

Recently, asymmetric supercapacitors (ASCs) have been regarded as promising due to their enhanced energy density. ASCs commonly combine pseudocapacitive materials (as positive electrode) and electric double-layer capacitive materials (as negative electrode), making use of the much higher specific capacitance derived from the pseudocapacitive electrode materials and a wider potential window during operation. In this sense, the electrochemical performances of ASCs are largely influenced by the structures and properties of the pseudocapacitive electrode materials. Thus, the development of high energy density ASCs heavily relies on the rational selection and design, as well as delicate fabrication of advanced electrode materials.

The use of transition metal oxides (TMOs) [1], hydroxides [2], sulfides [3], selenides [4], carbides [5], and their composites with conductive carbon and polymers [6], as possible electrode materials for ASCs, has been attempted. Particularly, TMOs, such as NiO [7], MoO_3_ [8], Co_3_O_4_ [9], and MnO_2_ [10], have received considerable attention due to their low cost, convenience of synthesis, environmental friendliness, and relatively high capacitance. Compared to binary TMOs, ternary TMOs materials containing two different metal cations, such as NiCo_2_O_4_ [11], ZnCo_2_O_4_ [12,13], CoMoO_4_ [14], MnMoO_4_ [15], and NiMoO_4_ [16], as well as some solid solutions [17], exhibit higher electrochemical activity due to the rich redox reactions stemming from the multiple oxidation states of the transition-metal components. It has been proposed that NiMoO_4_ has a good reversible capacitance and electrochemical characteristics for SCs/ASCs applications because of the electrochemical active Ni cation and improved electronic conductivity from Mo cation [16,18].

However, the practical application of NiMoO_4_ electrodes in SCs and ASCs is still hampered by their poor electronic conductivity, insufficient ionic transport and diffusion, and structural instability during long-term cycling [19]. Therefore, the controllable fabrication of NiMoO_4_ with desired nano- and microstructures and rational structural engineering is highly desired but remains challenging.

Various nanostructured NiMoO_4_ materials, such as nanosheets and nanorods arrays [20], nanotubes [21], hollow nanorods [22], mesoporous nanospheres [23], nanoparticles, and quantum dots [24], have been designed to boost the electrochemical performances of the NiMoO_4_ electrodes via increased exposed surface for ion adsorption and insertion, shortened path distances for ion transport and diffusion, and improved electrolyte impregnation and permeation. Specifically, various low-dimensional NiMoO_4_ nanostructures directly grown on conductive substrates (e.g., Ni/Cu foams [25,26], graphene [27], and carbon substrates [28,29]) are particularly preferred for directional electron transport with reduced charge carrier scattering at grain boundaries and easy integration into flexible devices with some specific applications.

To overcome the poor electronic conductivity of pristine NiMoO_4_, various NiMoO_4_/carbon composites have been synthesized by hybridizing NiMoO_4_ nanostructures with graphene [30,31], carbon nanotubes [32], conducting polymers [33], and porous carbon architectures [34,35] Alternatively, intentional doping of NiMoO_4_ with several kinds of heteroatoms such as Mn [36,37], P [38], Zn [39], Ce [40], or the creation of oxygen vacancies [14,41,42,43] in the lattice have recently been reported. In addition, NiMoO_4_ has also been coupled with other metal oxides [44,45,46,47,48,49] or sulfides [50,51,52] to form heterostructure electrodes for supercapacitors with improved electrochemical performances.

However, monotonous strategy sometimes has a limited contribution for the overall electrochemical performance improvement of NiMoO_4_ materials. In addition, some reported approaches for hybridization or doping of NiMoO_4_ involve multiple and complex chemical and physical processes that are not economically or environmentally friendly. Thus, the rational design and the design of a NiMoO_4_-based composite electrode for high-performance supercapacitors remains a challenge.

In this work, we report the simultaneous defect and interface engineering of NiMoO_4_ nanowires arrays (NWAs) using a simple and effective sucrose coating followed by a thermal treatment approach. In this process, an amorphous carbon shell was uniformly coated on the NiMoO_4_ surface, effectively improving the electronic transport and structural integrity of the NiMoO_4_ during electrochemical cycling. Additionally, oxygen-vacancy defects were incorporated into the NiMoO_4_ during the carbonization process, further enhancing the electronic conductivity of NiMoO_4_ and redox activity in the NiMoO_4_ electrode surface. As expected, the resultant NiMoO_4−*x*_@C composite exhibited a higher specific capacitance than that of the pristine NiMoO_4_ NWAs. Furthermore, an asymmetric supercapacitor (ASC) was assembled with the NiMoO_4−*x*_@C as positive electrode and activated carbon (AC) as negative electrode, delivering a remarkably high energy density of 51.6 W h kg^−1^ at a power density of 203 W kg^−1^ and an excellent cycling stability with a retention of 84.5% after 6000 cycles under a high current density of 10 A g^−1^.

## 2. Results and Discussion

**Figure 1:**
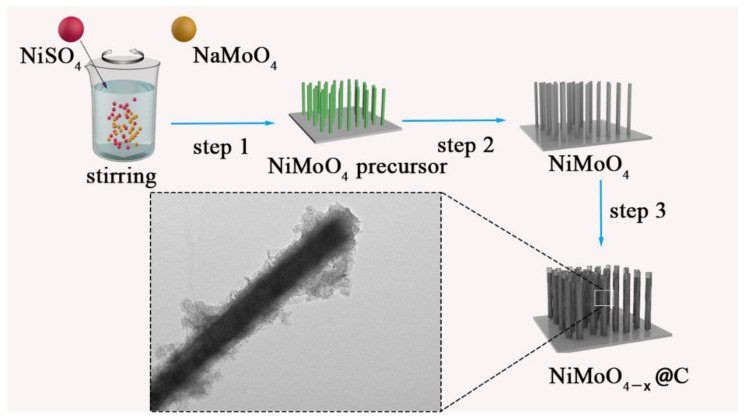
Schematic illustration of the synthesis process of oxygen-deficient NiMoO_4_@carbon nanowire arrays (NWAs) (denoted as “NiMoO_4−*x*_@C”). Step 1, growth of the NiMoO_4_ NWAs precursor directly on a ferronickel foam substrate using a hydrothermal process; Step 2, conversion of the NiMoO_4_ NWAs precursor into the NiMoO_4_ NWAs via annealing in Ar; Step 3, fabrication of NiMoO_4−*x*_@C composite by sucrose coating followed by annealing in Ar.

The synthesis route of oxygen-deficient NiMoO_4_@carbon nanowire arrays (NiMoO_4−*x*_@C) is schematically shown in Figure 1. The preparation process mainly involved three critical steps. Firstly, a NiMoO_4_ nanowire arrays (NWAs) precursor (NiMoO_4_·*x*H_2_O NWAs, light green) was directly deposited on a ferronickel foam by a hydrothermal reaction process (Step 1, Figure 1). Secondly, the NiMoO_4_ NWAs precursor was transferred into NiMoO_4_ NWAs by annealing in Ar to remove crystal H_2_O and improve crystallinity (Step 2, Figure 1). Finally, the as-obtained NiMoO_4_ NWAs were immersed in a sucrose solution, followed by drying and annealing in an Ar atmosphere to fabricate oxygen-deficient NiMoO_4_@carbon NWAs (NiMoO_4−*x*_@C) (Step 3, Figure 1).

**Figure 2:**
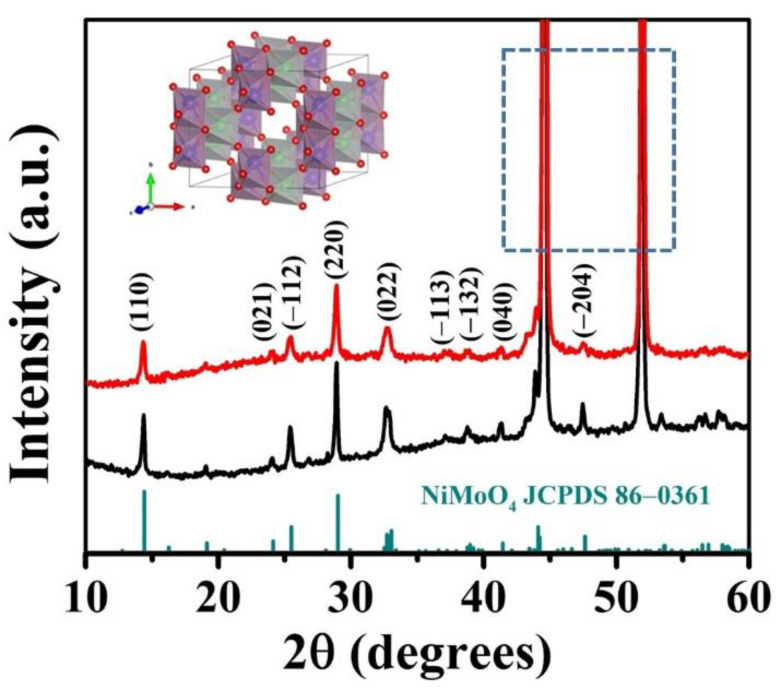
XRD patterns of NiMoO_4_ NWAs (black) and NiMoO_4__−*x*_@C composite (red). The inset (top left) shows the structural model of NiMoO_4_ crystal, whereas the green, blue, and red balls represent the Ni, Mo, and O atoms, respectively. The two strongest diffraction peaks in the square regions of dotted line are from the ferronickel foam substrate.

The crystal structures of NiMoO_4−*x*_@C and neat NiMoO_4_ NWAs samples were characterized by X-ray diffraction (XRD) analysis as depicted in Figure 2. The two strongest diffraction peaks, located at ca. 45° and 52°, were from the ferronickel foam substrate. The NiMoO_4−*x*_@C and NiMoO_4_ NWAs samples showed similar diffraction peak shapes and locations. The diffraction peaks located at 14.3°, 24.0°, 25.4°, 28.9°, 32.6°, 37.1°, 38.7°, 41.3°, and 47.4° corresponded to the (110), (021), (−112), (220), (022), (−113), (−132), (040), and (−204) crystal planes of orthorhombic NiMoO_4_ (JCPDS card No. 86-0361) [37]. Compared to the NiMoO_4_ NWAs samples, the NiMoO_4−*x*_@C sample exhibited a slightly lower diffraction peak intensity possibly due to the covering of carbon on the NiMoO_4_ surface as well as the reduced crystallinity of NiMoO_4_ with increased structural defects. In addition, no characteristic peaks for carbon phases were noted, suggesting the amorphous nature of the carbon species in the NiMoO_4−*x*_@C sample. The formation of amorphous carbon could be partially ascribed to the lower low annealing temperature (400 °C herein).

**Figure 3:**
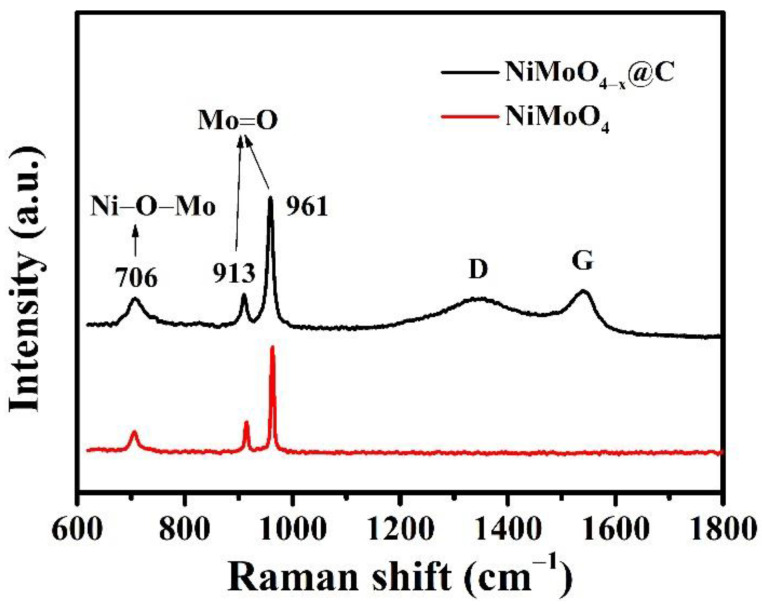
Raman spectra of NiMoO_4−*x*_@C (black) and pristine NiMoO_4_ NWAs (red) samples.

Raman spectra of pristine NiMoO_4_ NWAs and NiMoO_4−*x*_@C samples are illustrated in Figure 3. The bands at 961 cm^−1^ and 913 cm^−1^ corresponded to the symmetric and asymmetric stretching modes of Mo=O bonds, while the band at 706 cm^−1^ could be ascribed to the stretching mode of Ni/Mo-O bonds of the orthorhombic α-NiMoO_4_ phase [53]. In addition, two bands ascribed to the presence of carbon species were identified. The band at around 1360 cm^−1^ could be attributed to the D band from defects and disorders in the amorphous carbon layers, while the other band at around 1590 cm^−1^ was related to the G band related to the vibration of sp^2^-bonded carbon atoms [54]. This result implied the successful deposition of amorphous carbon layer on the surface of the NiMoO_4_ NWAs.

**Figure 4:**
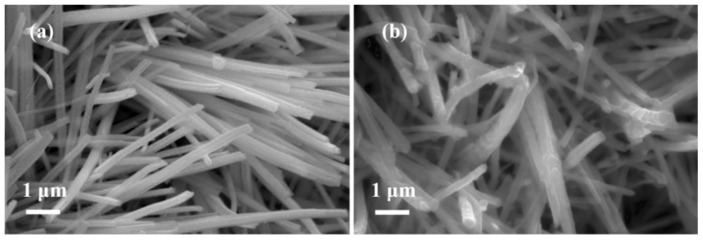
SEM images of (**a**) neat NiMoO_4_ NWAs and (**b**) NiMoO_4−x_@C samples.

The morphologies of the NiMoO_4_ samples were firstly observed using scanning electron microscopy (SEM). From Figure 4a, the pristine NiMoO_4_ NWAs sample was composed of oriented nanowires (NWs) with a smooth surface. In addition, the NiMoO_4_ NWAs have relatively uniform diameters of ~300 nm, on average, and lengths of several micrometers. After the coating of the carbon, the surface of the NiMoO_4−*x*_@C sample became obviously coarse as shown in Figure 4b. The element composition analyses using energy-dispersive X-ray spectra (EDS) analysis indicated the existence of Ni, Mo, O, C, Fe, and Al elements in the NiMoO_4−*x*_@C sample (Figure S1, Supporting Information). Note that the Fe and Al signals mainly stemmed from the ferronickel foam substrate and the sample holder, respectively.

**Figure 5:**
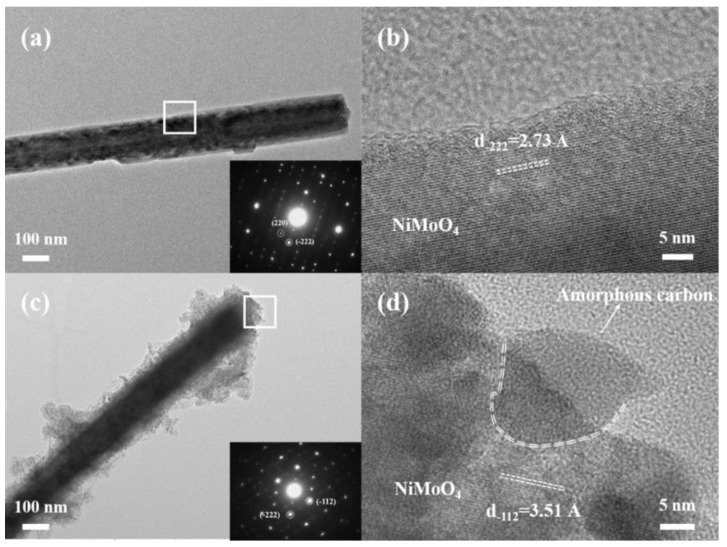
TEM images (**a**,**c**) and HRTEM micrographs (**b**,**d**) of pure NiMoO_4_ NWAs (**a**,**b**) and NiMoO_4−*x*_@C (**c**,**d**) samples. The insets in (**a**,**c**) are corresponding SAED patterns taken from a single nanowire.

The microstructures of the pure NiMoO_4_ NWAs and NiMoO_4−*x*_@C samples were further investigated by transmission electron microscope (TEM) and high-resolution TEM (HRTEM) as shown in Figure 5. The TEM image (Figure 5a) revealed that the pure NiMoO_4_ nanowire had a smooth surface with a diameter of about 200 nm. A selected-area electron diffraction (SAED) pattern (inset of Figure 5a) taken from this nanowire depicted a clear two-dimensional dot pattern, suggesting its single-crystalline structure in nature. Two diffraction spots, as marked by white circles, could be indexed to the (220) and (−222) crystal facets of orthorhombic NiMoO_4_. From Figure 5b, the crystal plane with a lattice spacing of 2.73 Å in the HRTEM micrograph corresponded to the (−222) planes of NiMoO_4_ [49,52]. In contrast, the TEM image in Figure 5c indicated that a layer of amorphous carbon film with a thickness of ca. 20~50 nm had been coated on the NiMoO_4_ nanowire’s surface, confirming the core-shell structure of the NiMoO_4−*x*_@C composite sample with different brightness contrasts of NiMoO_4_ and carbon. The deposition of amorphous carbon on the surface of NiMoO_4_ can be further confirmed by HRTEM micrograph as shown in Figure 5d.

**Figure 6:**
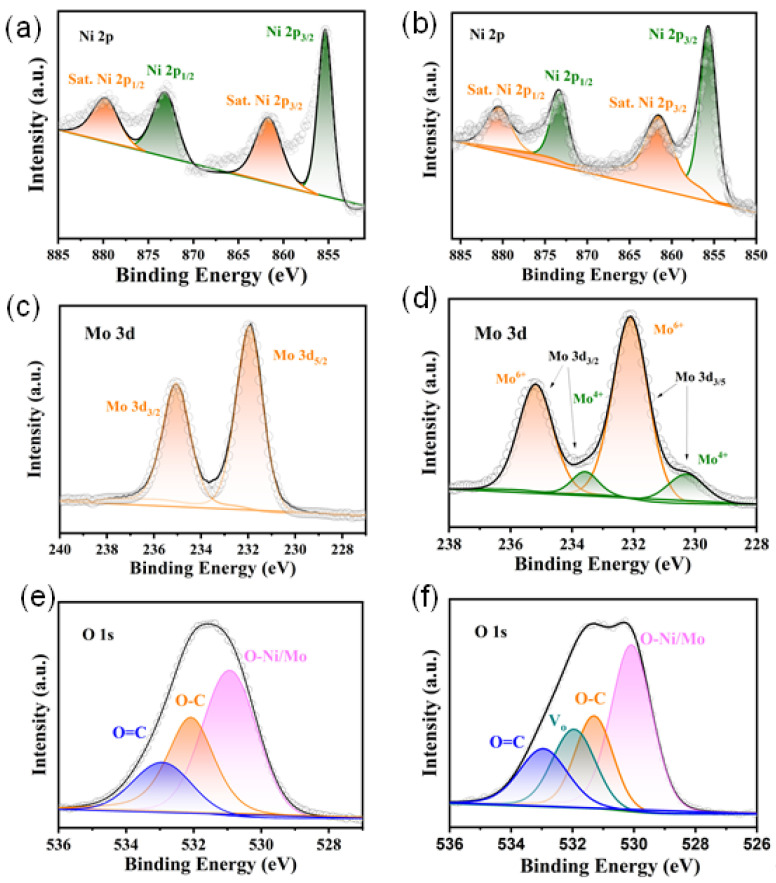
High-resolution XPS spectra of (**a**,**c**,**e**) pristine NiMoO_4_ and (**b**,**d**,**f**) NiMoO_4−*x*_@C samples; (**a**,**b**) Ni 2p, (**c**,**d**) Mo 3d, (**e**,**f**) O 1s.

Next, the chemical composition and valence states of element on the surface of NiMoO_4_ NWAs and NiMoO_4−*x*_@C samples were identified by X-ray photoelectron spectroscopy (XPS, Figure 6). In the high-resolution Ni 2p spectrum of the pristine NiMoO_4_ NWAs sample (Figure 6a), two main peaks were observed at binding energies (BEs) of 873.4 eV and 856.3 eV with a spin-orbital splitting energy of 17.1 eV, corresponding to the Ni 2p_1/2_ and Ni 2p_3/2_ of Ni^2+^ in NiMoO_4_ lattice [55]. In addition, two satellite peaks with BEs of 877.3 eV and 860.6 eV were noted for Ni^2+^. In Figure 6b, similar peak locations and separations can also be observed in the Ni 2p spectra of NiMoO_4−*x*_@C sample, indicating the coating of carbon had little effect on the chemical valence states of the Ni component in NiMoO_4_. The Mo 6d spectrum of pure NiMoO_4_ and NiMoO_4−*x*_@C samples are shown in Figure 6c,d. Evidently both samples had two strong bands with BEs located at 236.0 eV and 232.9 eV, which could be assigned to Mo 3d_3/2_ and Mo 3d_5/2_ of Mo^6+^ cations in the NiMoO_4_ lattice [56]. In addition, another pair of doublets was noted for the NiMoO_4−*x*_@C sample, verifying the existence of Mo^4+^ in the NiMoO_4−*x*_@C composite [41,42,57] possibly produced during the amorphous carbon coating process. From Figure 6e, the O 1s spectrum of the NiMoO_4_ NWs sample was deconvoluted into three bands. The band centered at 530.1 eV was assigned to the lattice oxygen with O-Ni/O-Mo bonds, while the bands located at 531.3 eV and 532.9 eV correspond to the O-C and O=C bonds from moisture adsorbed on surface [58]. For the NiMoO_4−*x*_@C sample, another band could be noted at 532 eV, suggesting the presence of oxygen vacancies (V_o_) [59] at the NiMoO_4_ surface (Figure 6f). The formation of and Mo^4+^ and V_o_ can tune the electronic structures and electrochemical properties of the NiMoO_4−*x*_@C composite sample.

Then, the effects of pyrolysis temperatures (from 200~800 °C) during the carbon coating of the morphologies and microstructures of the NiMoO_4_/carbon composites were investigated. It is noted that some aggregates of residual sucrose were observed after annealing at 200 °C (Figure S2a), suggesting the carbonization of sucrose was incomplete under a lower temperature. This SEM result also coincides well with the thermogravimetric (TGA) and the differential scanning calorimetry (DSC) analyses (Figure S3), where the thermal decomposition process of sucrose mainly occurs between 223 and 389 °C. With the increase of annealing temperature, sucrose was decomposed, and the carbonization process occurred accompanied by the release of some gases (e.g., CO, CO_2_). At a higher temperature, the generated reductive gases (e.g., CO) reacted with NiMoO_4_ and generated some oxygen vacancies on the NiMoO_4_ surface via abstracting some surface oxygen atoms. In contrast, well-defined nanowires were obtained for the samples prepared after annealing at 400 and 600 °C, respectively (Figure S2b,c). However, the nanowire structure was destroyed when the pyrolysis temperature was increased to 800 °C (Figure S2d), which might have been caused by the large inner stain in the NiMoO_4_ NWAs or at the NiMoO_4−*x*_@C interface. Thus, the standard annealing temperature was chosen as 400 °C.

**Figure 7:**
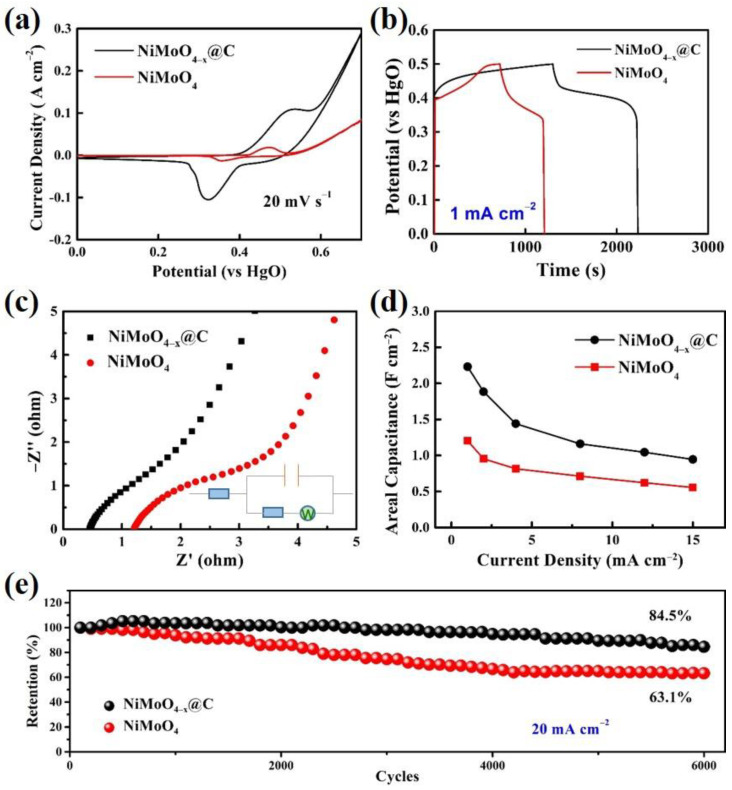
(**a**) CV curves of the NiMoO_4_ NWAs and NiMoO_4−*x*_@C at a scan rate of 20 mV s^−1^. (**b**) GCD curves and (**c**) EIS spectra of NiMoO_4_ NWAs and NiMoO_4−*x*_@C and its corresponding equivalent fitting circuit. (**d**) GCD test of NiMoO_4−*x*_@C at different current densities. (**e**) Cycle test of NiMoO_4_ NWAs and NiMoO_4−*x*_@C at 20 mA cm^−2^ over 6000 cycles.

To evaluate the electrochemical performance of NiMoO_4_ NWAs and NiMoO_4−*x*_@C samples, electrochemical measurements were tested by a three-electrode system with 2 M KOH electrolyte (Figure 7). Figure 7a shows the CV curves of NiMoO_4_ NWAs and NiMoO_4−*x*_@C samples at a scan rate of 20 mV s^−1^ with a potential window of 0 to 0.7 V. Overall, the NiMoO_4−*x*_@C sample had a larger integral area than that of the NiMoO_4_ NWAs sample, indicating a significant increase of capacitance after carbon deposition and introduction of oxygen vacancies. Meanwhile, the CV curves of the two samples exhibited typical oxidation peaks, demonstrating typical pseudocapacitive charge storage characteristics. In addition, the CV curves of four different pyrolysis temperatures of NiMoO_4−*x*_@C samples are revealed in Figure S4. The sample collected at 400 °C shows the highest capacitance which is consistent with the result of SEM in Figure S2. The sample collected at 800 °C exhibited an unsatisfactory performance due to its collapsed morphology. Figure 7b shows the GCD curves of the two samples. It revealed that the discharge time of the NiMoO_4−*x*_@C sample was almost twice as much as that of the NiMoO_4_ NWAs sample at a current density of 1 A cm^−2^. Figure 7c shows the electrochemical impedance spectroscopy (EIS) of NiMoO_4_ NWAs and NiMoO_4−*x*_@C. The direct impedance and charge transfer resistance of the NiMoO_4−*x*_@C sample was significantly lower than that of the NiMoO_4_ NWAs sample. The remarkably reduced size of the semicircle for the NiMoO_4−*x*_@C indicated an improved charge transfer kinetics due to enhanced electrical conductivity provided by the carbon shell and oxygen vacancy defects. In addition, the NiMoO_4−*x*_@C exhibits the steepest slope in the low-frequency region, clearly indicating the lowest Warburg impedance and, hence, the highest K-ion diffusion capability at the interface between the electrode and electrolyte. Through the AC EIS, we added the corresponding equivalent circuit diagram in Figure 7c.

The true impedance of capacitor can be estimated using the following Equation (1):

(1) $$Z_{real}=R_{\Omega }+\frac{-j\left(wC\right)\left(R_{ct}+W\cdot jw^{1/2}\right)}{R_{ct}+W\cdot jw^{1/2}-j\left(wC\right)}$$

Figure 7d shows the capacitance of NiMoO_4_ NWAs and NiMoO_4−*x*_@C samples calculated from different current densities. After coating the carbon layer, the capacitance of the NiMoO_4−*x*_@C sample was greatly increased. The areal capacitance can be calculated as high as 2.24 F cm^−2^ (1720 F g^−1^) at a current density of 1 mA cm^−2^. In contrast, the NiMoO_4_ NWAs electrode only demonstrated a specific capacitance of 1.206 F cm^−2^ (927 F g^−1^) at the same current density. The cycling performances of NiMoO_4_ NWAs and NiMoO_4−*x*_@C samples are presented in Figure 7e. The capacitance retention of NiMoO_4−*x*_@C is 84.5% at 20 mA cm^−2^ after 6000 cycles, which is considerably better than that of the NiMoO_4_ NWAs sample (63.1% after 6000 cycles). To illustrate the difference of the cycling process between NiMoO_4_ NWAs and NiMoO_4−*x*_@C samples, we also obtained the SEM results after cycling as shown in Figure S5. It is evident that the NiMoO_4−*x*_@C sample still held some nanorod structures under the protection of amorphous carbon shell. Instead, NiMoO_4_ NWAs were aggregated after 10,000 cycles, with unsatisfactory cycle abilities. In addition, we made a comparison of the C_s_ and cycling stability of this work with some previously reported NiMoO_4_-based electrodes materials as summarized in Table S1.

**Figure 8:**
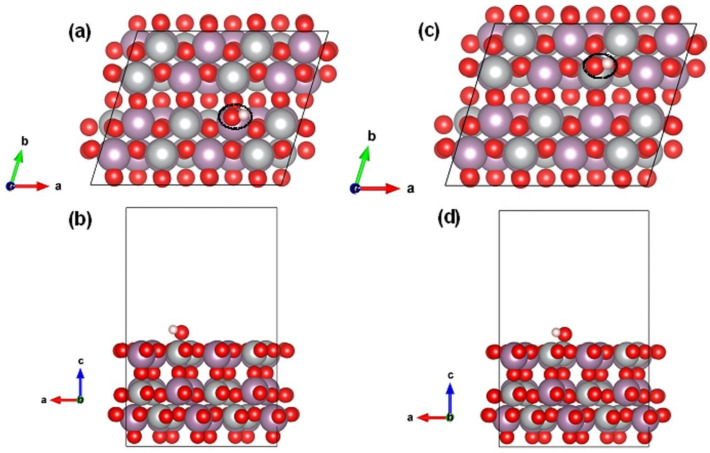
The optimized adsorption configurations of one HO molecule anchored on exposed (**a**,**b**) Mo- and (**c**,**d**) Ni- atoms of pristine NiMoO_4_ (110) surfaces, leading to an *E*_ads_ of −0.50 and −5.97 eV, respectively. The black dotted ellipsoids in (**a**,**c**) mark the positions of the adsorbed HO molecule on the NiMoO_4_ (110) surface.

First-principles density functional theory (DFT) simulations were next adopted to further probe the structure–performance relationship of the NiMoO_4−*x*_@C composite electrode in supercapacitors. The optimized geometry configurations of pristine and oxygen-deficient NiMoO_4_ (110) surface slabs are shown in Figure S6. The pristine NiMoO_4_ (110) surface was flat and composed of fivefold Ni and Mo atoms and twofold O atoms (Figure S6a). The defective NiMoO_4_ (110) surface can be produced after eliminating one surface O atom, leaving one threefold Ni and Mo atoms nearby (Figure S6b). The resultant oxygen-deficient NiMoO_4_ (110) plane retains flat. Next, the adsorption behavior of OH group on the pristine NiMoO_4_ (110) surface was first investigated. As shown in Figure 8, the OH can be adsorbed on the top of either the surface of the Mo atom (Figure 8a,b) or the Ni atom (Figure 8c,d), yielding an adsorption energy (*E*_ads_) of −0.50 and −5.97 eV, respectively. Evidently, the adsorption of OH on the Ni site was much stronger than that on the Mo site, which is consistent with the fact that the Ni in NiMoO_4_ is electrochemically active for pseudocapacitive charge storage process based on Faradic reactions. The chemisorption of OH on Ni and Mo sites of the NiMoO_4_ (110) surface was further verified by the interfacial charge transfer from the charge density difference contours (Figure S7).

**Figure 9:**
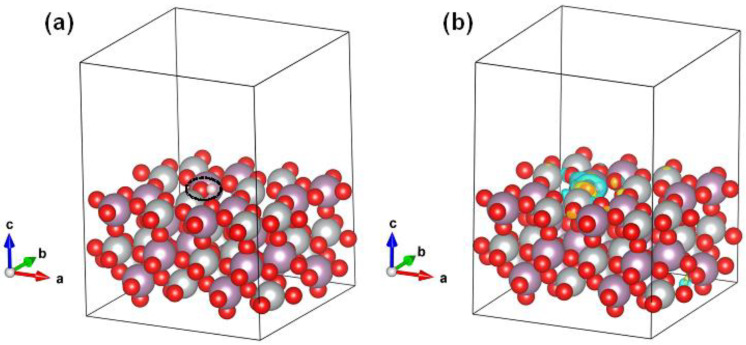
The optimized adsorption configurations of one HO molecule anchored on NiMoO_4_ (110) surfaces with one surface oxygen vacancy (V_o_), leading to an *E*_ads_ of −3.63 eV. The black dotted ellipsoid in (**a**) labels the position of the adsorbed HO molecule, which is at the vicinity of the V_o_. The yellow and cyan colors in the charge density difference contour (**b**) represent the electron accumulation and depletion, respectively. The isosurface level is 0.001 e bohr^−3^.

In the following, the adsorption of OH adsorbed on the oxygen-deficient NiMoO_4_ (110) surface was evaluated. Specifically, the adsorption on the Ni and Mo sites with lower coordination due to the removal of surface O was considered. Interestingly, it is noted that the OH group was preferred to be adsorbed at the vicinity of the oxygen-vacancy position (Figure 9a), leading to an *E*_ads_ of −3.63 eV and a charge transfer at the OH/NiMoO_4_ interface (Figure 9b). This result suggests that the presence of surface oxygen vacancies offers more active sites for OH adsorption, concentration, and subsequent redox reactions for enhanced pseudocapacitive charge storage.

Based on the above experimental data and theoretical simulations, the significantly improved electrochemical performances of the NiMoO_4−*x*_@C composite can be mainly attributed to the following points: (i) the deposition of amorphous carbon shell effectively enhances the electron transport of NiMoO_4_ nanowires and charge transfer at NiMoO_4_/C heterointerface; (ii) the deposited carbon layer also improves the structural integrity of the NiMoO_4_ nanowire arrays during long-term electrochemical cycling; (iii) the creation of oxygen vacancies in NiMoO_4_ accompanied by the coating of the carbon further enhances the electronic conductivity of the NiMoO_4_ electrode and creates more active sites for pseudocapacitive charge storage. Therefore, the synergy of defect and interface engineering of NiMoO_4_ NWAs realized by carbon deposition effectively improves the overall electrochemical performance of the resultant NiMoO_4−*x*_@C composite electrode in supercapacitors.

**Figure 10:**
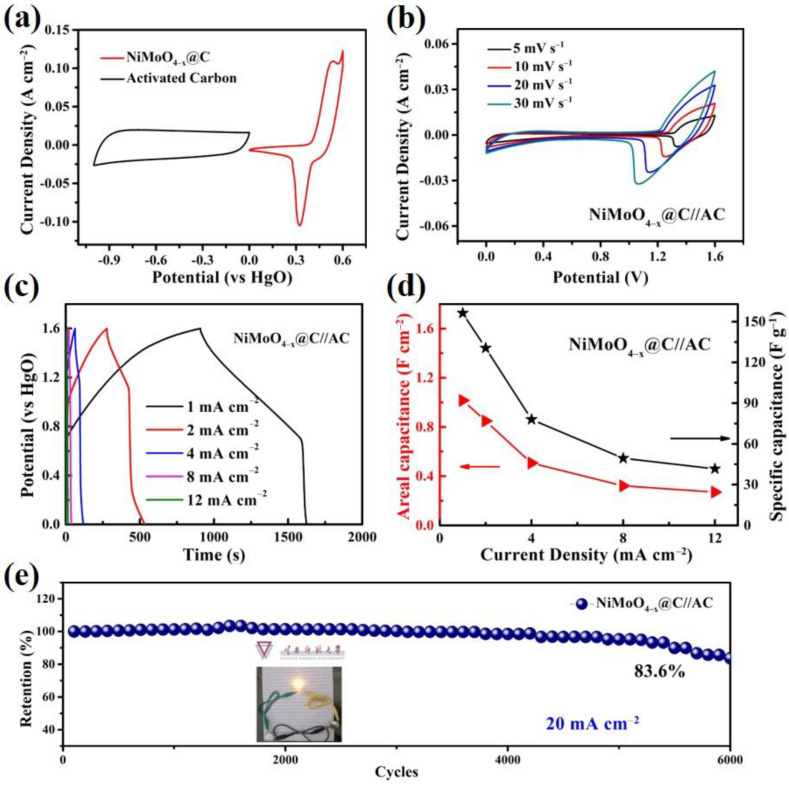
Electrochemical performance of the asymmetric NiMoO_4−*x*_@C//AC supercapacitor. (**a**) CV curves of activated carbon (AC) and NiMoO_4−*x*_@C electrodes from −1.0~0 V and 0~0.6 V, respectively at 5 m V s^−1^; (**b**) CV curves; (**c**) GCD curves; (**d**) areal capacitance and specific capacitance; (**e**) cyclic stability of NiMoO_4−*x*_@C//AC.

To further assess the practical application potential of a NiMoO_4−*x*_@C sample, an asymmetric supercapacitor device (NiMoO_4−*x*_@C//AC) was assembled with the NiMoO_4−*x*_@C as a positive electrode and activated carbon (AC) as a negative electrode. Before testing the ASC device, we performed the CV measurements of NiMoO_4−*x*_@C and AC electrodes in a three-electrode system at a scan rate of 5 mV s^−1^ to estimate the suitable operating voltage range (Figure 10a). The maximum operating voltage of NiMoO_4−*x*_@C//AC ASC was determined to be 1.6 V. The CV curves of NiMoO_4−*x*_@C//AC ASC at different scan rates are shown in Figure 10b. All the curves display obvious redox peaks, indicating the main contribution from pseudocapacitance. An increased separation of redox peak position can be noted along with the increase of scan rates due to the increased polarization. Figure 10c shows the GCD curves of the NiMoO_4−*x*_@C//AC ASC at different current densities while Figure 10d shows the specific capacitance calculated from different current densities. The overall capacitance of the ASC was calculated to be 1.01 F cm^−2^ (156.25 F g^−1^) at 1 mA cm^−2^. Furthermore, the NiMoO_4−*x*_@C//AC ASC device has demonstrated a good capacitance retention of 83.6% after 6000 cycles at 20 mA cm^−2^ (Figure 10e). As a result, the ASC device can power a yellow LED (inset of Figure 10e), showing its potential in practical applications. In addition, the electrochemical performances of our NiMoO_4−*x*_@C//AC ASC device are also superior or comparable to some recently reported NiMoO_4_-based electrode materials for ASCs as summarized in Table S1.

## 3. Conclusions

In summary, synchronous defect and interface engineering was implemented in NiMoO_4_ material via the formation of oxygen vacancies and the coating of the carbon on NiMoO_4_ nanowire arrays through a simple hydrothermal method paired with sucrose pyrolysis. During this process, an amorphous carbon layer was homogeneously deposited on the surface of NiMoO_4_ nanowires and oxygen vacancies were created on the NiMoO_4_ surface during the carbonization of sucrose. The deposited carbon layer and formed oxygen vacancies in NiMoO_4_ boosted the electronic conductivity of NiMoO_4_ nanowires. In addition, the coated carbon layer also improved the structural integrity of the NiMoO_4_ electrode during long-term operation in supercapacitors. Consequently, the resultant NiMoO_4−*x*_@C heterostructure electrode achieved a high specific capacitance of 2.24 F cm^−2^ (1720 F g^−1^) and maintained a good capacitance retention of about 83.6% after 6000 cycles at 20 mA cm^−2^. In addition, the as-assembled NiMoO_4−*x*_@C//activated carbon asymmetric supercapacitor device manifested a high energy density of 51.6 W h kg^−1^ at a high power density of 203.95 W kg^−1^, indicating that NiMoO_4−*x*_@C composite is a suitable electrode material for supercapacitor applications. The proposed synergistic defect and interface engineering strategy herein can be extended for the design and development of other novel composite electrode materials for applications in electrochemical energy storage and conversion.

## Data Availability

All data included in this study are available upon request by contact with the corresponding author.

## Supplementary Materials

The following supporting information can be downloaded at https://www.mdpi.com/article/10.3390/nano12071094/s1, Figure S1: The EDS analysis of NiMoO_4−*x*_@C composite and NiMoO_4_ NWAs, Figure S2: SEM images of the NiMoO_4−*x*_@C composite prepared under different annealing temperature: (a) 200 °C, (b) 400 °C, (c) 600 °C and (d) 800 °C, Figure S3: Thermogravimetric (TG) and differential scanning calorimetry (DSC) analysis of sucrose in Ar atmosphere at a ramping rate of 10 °C min^−1^, Figure S4: (a) CV and (b) GCD of NiMoO_4−*x*_@C samples prepared under different annealing temperatures, Figure S5: SEM images of the (a) NiMoO_4−*x*_@C and (b) NiMoO_4_ NWAs electrodes after 10,000 cycles at 20 mA cm^−2^, Figure S6: Optimized geometry structures of the (a) pristine NiMoO_4_ (110) surface and (b) oxygen-deficient NiMoO_4_ (110) surface obtained by removing one surface O atom (green). The grey, light pink and red balls represent the Ni, Mo and O atoms, respectively [60,61,62,63], Figure S7: Charge density difference contours of after adsorption of one HO molecule on (a) Mo- and (b) Ni-side of NiMoO_4_ (110) surface, respectively. The yellow and cyan colors denote the electron gain and loss, respectively. The isosurface level is 0.001 e bohr^−3^, Table S1: Brief comparison of electrochemical performance of current work with recently relevant literature [20,22,30,55,64,65,66,67,68,69,70,71,72].
