# Supplementary material for: Synchronous Defect and Interface Engineering of NiMoO4 Nanowire Arrays for High-Performance Supercapacitors
Source: Nanomaterials (Basel). 2022 Mar 26;12(7):1094. doi: 10.3390/nano12071094 (PMC9000437; doi:10.3390/nano12071094)
Supplement: Supplementary file 1 [file nanomaterials-12-01094-s001.zip › nanomaterials-1639888-supplementary.pdf]

nanomaterials

MDPI

Supplementary Materials

Synchronous Defect and Interface Engineering of $NiMoO_4$ Nanowire Arrays for High-Performance Supercapacitors

Pengcheng Wang [1], Xinying Ding [1], Rongjie Zhe [1], Ting Zhu [1,2], Chen Qing [1,2,*], Yingkai Liu [1] and Hong-En Wang [1,2,*]

[1] Yunnan Key Laboratory of Optoelectronic Information Technology, College of Physics and Electronics Information, Yunnan Normal University, Kunming 650500, China; pcwang0312@163.com (P.W.); xinyingding1998@163.com (X.D.); rongjiezhe@163.com (R.Z.); zhut0002@ynnu.edu.cn (T.Z.); ykliu@ynnu.edu.cn (Y.L.)

[2] Key Laboratory of Advanced Technique & Preparation for Renewable Energy Materials, Ministry of Education, Yunnan Normal University, Kunming 650500, China

* Correspondence: qingchen1@126.com (C.Q.); hongenwang@whut.edu.cn (H.-E.W.)

EXPERIMENTAL SECTION

Preparation of $NiMoO_4$ nanowire arrays (NWAs)

All the reagents were analytical grade and used without further purification. The nickel sulfate ($NiSO_4{\cdot}6H_2O$) and sodium molybdate ($Na_2MoO_4{\cdot}2H_2O$) were obtained from Aladdin reagent (Shanghai) Corporation. The ferronickel foam substrate (5 × 2 cm^2) was immersed in 10wt.% hydrochloric acid solution to remove the surface oxide layer, followed by rinsing with distilled H_2O and drying at 60 °C for 1 h. 1.5 mmol of $NiSO_4{\cdot}6H_2O$ and 1.5 mmol of $Na_2MoO_4{\cdot}2H_2O$ were dissolved in 50 mL of distilled water under constant magnetic stirring for 10 min. Then, the resulting mixed solution was transferred into 100 mL Teflon-lined stainless-steel autoclave with pretreated ferronickel foam substrate inside. The autoclave was maintained at 150 ℃ for 6 h. After the hydrothermal reaction, the $NiMoO_4$ nanowire arrays (NWAs) precursor was cleaned by ultrasonic treatment in deionized water for 5 min to remove any loosely attached nanoparticles adsorbed on the surface. Finally, the $NiMoO_4$ NWAs precursor was annealed at 350 ℃ for 2 h in Ar with a heating rate of 10 ℃ min^{-1}. The average mass loading of $NiMoO_4$ NWAs was determined to be *ca.* 1.3 mg cm^{-2}.

Preparation of $NiMoO_{4-x}$@C composite

$NiMoO_{4-x}$@C composite was prepared by coating amorphous carbon shell on the surface of $NiMoO_4$ NWAs via thermal decomposition reaction of sucrose. In a typical procedure, the as-prepared $NiMoO_4$ NWAs (1.3 mg cm^{-2} × 10 cm^2 = 13 mg) were firstly immersed in 50 mL of 0.1 mol L^{-1} sucrose solution for 6 h, followed by drying at 60 °C in vacuum for 1 h. Then, the sucrose-coated $NiMoO_4$ NWAs were annealed at 400 °C for 2 h in Ar, followed by cooling to room temperature naturally to obtain the final $NiMoO_{4-x}$@C product. To study the effects of annealing temperature on the structure and electrochemical properties, the sucrose-coated $NiMoO_4$ NWAs were also calcined at 200 °C, 600 °C, and 800 °C, respectively, for comparison.

Characterizations

Scanning electron microscopy (SEM, Quanta250 FEG, 30 kV), transmission electron microscopy (TEM, JEM-2100, 200 kV), X-ray diffractometer (XRD, domestic DX-2700, Cu Kα radiation: λ = 1.5406 Å), X-ray photoelectron spectrometer (XPS, K-Alpha+), and Raman spectroscopy (LabRAM HR Evolution in HORIBA) were used to characterize the as-received samples. Cyclic voltammetry (CV), galvanostatic charge and discharge (GCD) and electrochemical impedance spectroscopy (EIS, frequency range of 100 kHz to 0.1 Hz) were tested by CHI660E electrochemical workstation at room temperature.

Electrochemical measurements

The electrochemical performance of $NiMoO_4$ NWAs and $NiMoO_{4-x}$@C were tested by using three-electrode system in a 2 M KOH aqueous solution. Hg/HgO electrode was used as reference electrode, platinum plate was used as counter electrode and $NiMoO_{4-x}$@C composite (or $NiMoO_4$ NWAs) electrode with a size of 1 × 1 cm^2 was used as the working electrode. For the two-electrode system, an asymmetric supercapacitor device ($NiMoO_{4-x}$@C//AC) was assembled with the $NiMoO_{4-x}$@C as positive electrode and activated carbon (AC) as negative electrode, respectively, 2 M KOH as the electrolyte and cellulose paper as the separator. According to the principle of charge balance following the relationship $q^+ = q^-$. The charge stored by each electrode depends on the specific capacitance (C), the potential range for the charge/discharge process (ΔE) and the mass of the electrode (m) following the equation:

$$q = C \times \Delta E \times m$$

and in order to get $q^+ = q^-$, the mass balancing follows the equation:

$$\frac{m^+}{m^-} = \frac{C_- \times \Delta E_-}{C_+ \times \Delta E_+}$$

the mass ratio of positive and negative electrodes is about 1:4.08. Based on the mass of $NiMoO_{4-x}$@C (1.3 mg), 5.3 mg of activated carbon was used to assemble the device, and thus the total mass of $NiMoO_4$/AC//ACNE is 6.6 mg. The areal capacitance was calculated from the discharge curve as follows: C = It / (ΔV*S), where C (F cm^{-2}) is the areal capacitance, I (A) is the discharge current, t (s) is the discharge time, ΔV (V) is the potential window, and S (cm^2) is the area of $NiMoO_4$/AC electrode. All the electrochemical tests were carried out at room temperature.

Computational details

Spin-polarized first-principles density functional theory (DFT) calculations were performed using projector augmented wave (PAW) pseudopotentials with an exchange-correlation functional of Perdew-Burke-Ernzerhof (PBE) formation as implemented in VASP [1,2]. An energy cutoff of 520 and 400 eV was adopted for $NiMoO_4$ conventional cell (containing 8 Ni atoms, 8 Mo atoms and 32 O atoms) and surface slabs, respectively. The k-point sampling was obtained using a Γ-centered Monkhorst-Pack scheme with a 3 × 3 × 4 mesh for conventional cell and 2 × 2 × 1 mesh for surface slabs, respectively. The convergence criteria for SCG iteration and force were set to 1.0 × 10^{-5} eV/atom and 0.015 eV/Å for $NiMoO_4$ conventional cells while 1.0 × 10^{-4} eV/atom and 0.05 eV/Å for surface slabs, respectively. The $NiMoO_4$ (110) surface slab consists of five atomic layers with 24 Ni atoms, 24 Mo atoms and 96 atoms, with a size a 15.52 Å × 12.50 Å × 23.41 Å which includes 15 Å vacuum layer along z-axis to avoid unwanted interactions among neighboring layers. To simulate the defective $NiMoO_4$ (110) surface, one surface O atom was removed from the surface slab. PBE+U method with U = 6.2 and 4.38 eV was added to the 3d orbitals of Ni and Mo, respectively to adjust the strength of Coulomb and exchange interactions.

The adsorption energies (Eads) were determined using the following equation:

$$E_{ads} = E_{mol/sub} - (E_{mol} + E_{sub})$$

where $E_{mol/sub}$, E_{mol} and E_{sub} denote the total electronic energies of $NiMoO_4$ surface with adsorbed OH, OH molecule in gas phase, and clean $NiMoO_4$ surface, respectively. During the surface calculations, dipole correction was adopted along *z*-axis and van der Waals interactions were considered using DFT-D method. The drawing of the calculated data was performed using VESTA [3]. Some pre- and post-treatment processes were carried out with the assistance of VASPKIT [4].

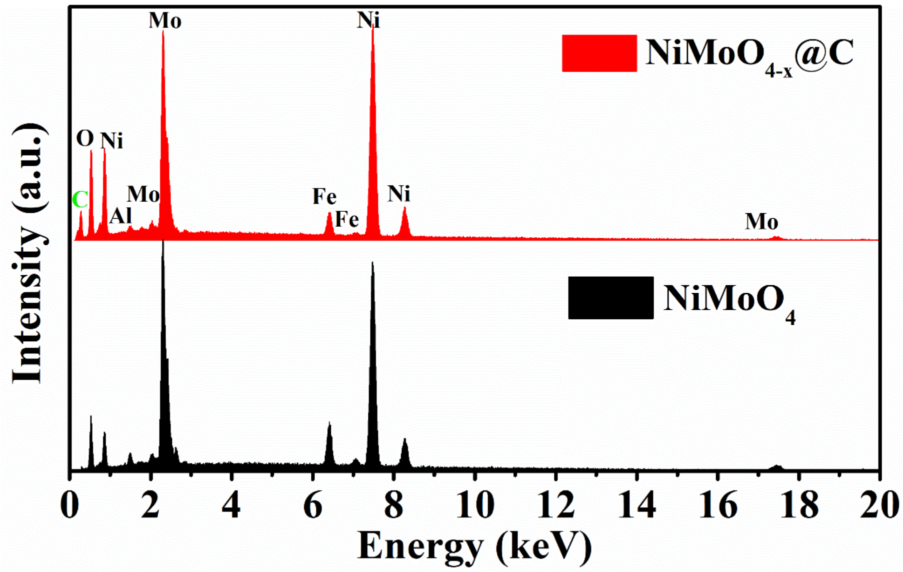

Figure S1. The EDS analysis of $NiMoO_{4-x}$@C composite and $NiMoO_4$ NWAs.

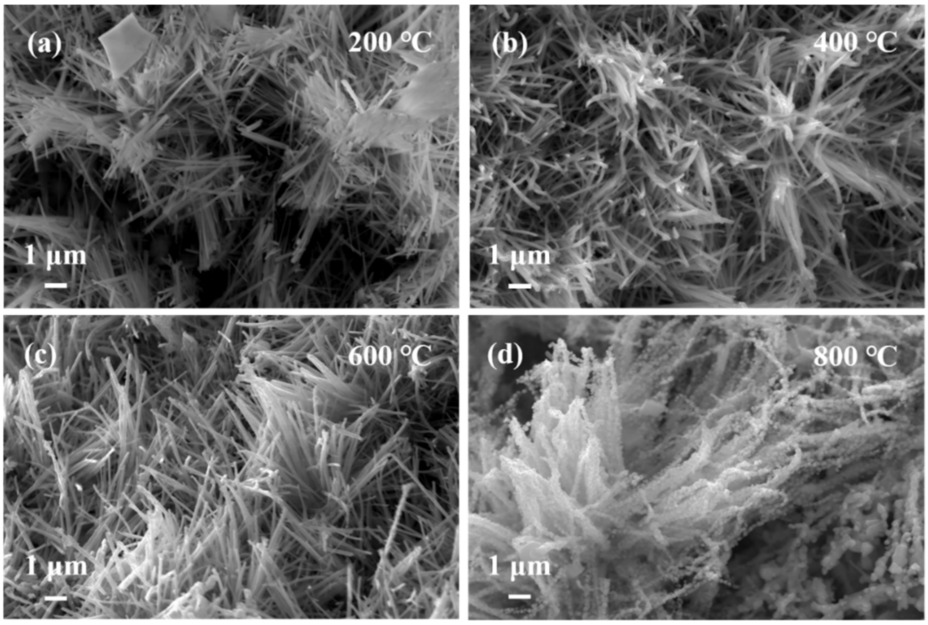

Figure S2. SEM images of the $NiMoO_{4-x}$@C composite prepared under different annealing temperature: (**a**) 200 °C, (**b**) 400 °C, (**c**) 600 °C and (**d**) 800 °C.

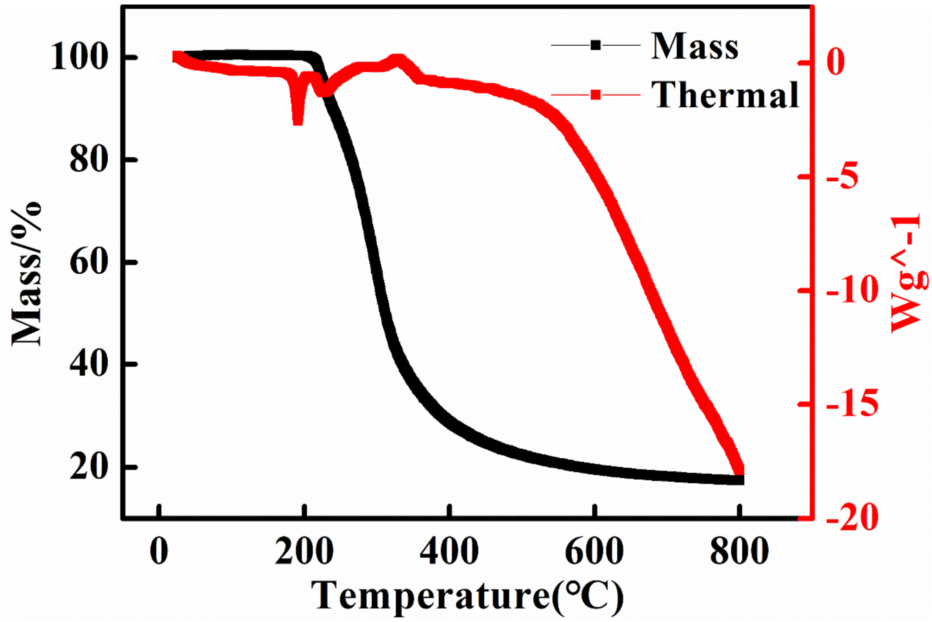

Figure S3. Thermogravimetric (TG) and differential scanning calorimetry (DSC) analysis of sucrose in Ar atmosphere at a ramping rate of 10 °C min^{-1}.

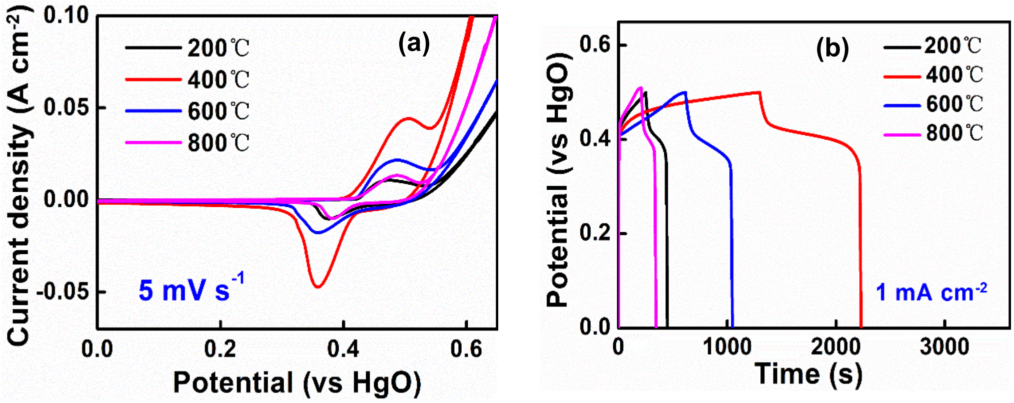

Figure S4. (**a**) CV and (**b**) GCD of $NiMoO_{4-x}$@C samples prepared under different annealing temperatures.

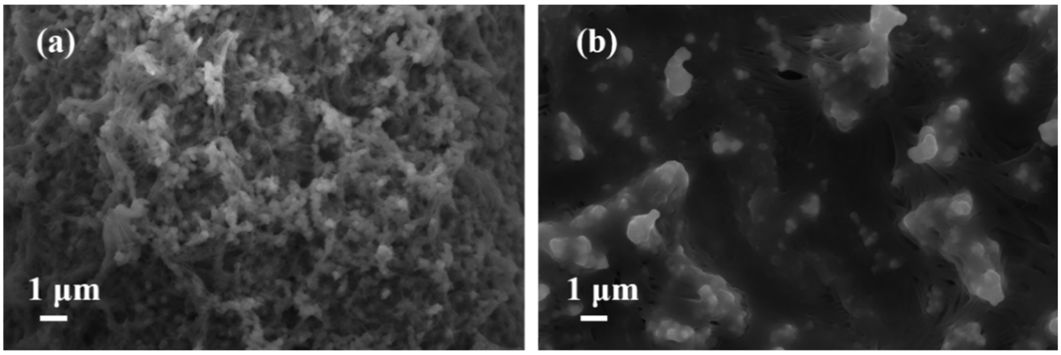

Figure S5. SEM images of the (**a**) $NiMoO_{4-x}$@C and (**b**) $NiMoO_4$ NWAs electrodes after 10,000 cycles at 20 mA cm^{-2}.

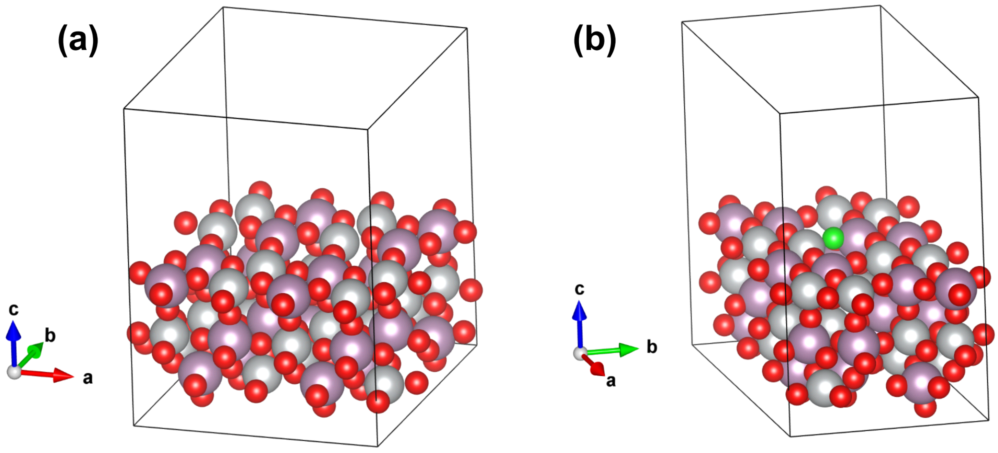

Figure S6. Optimized geometry structures of the (**a**) pristine $NiMoO_4$ (110) surface and (**b**) oxygen-deficient $NiMoO_4$ (110) surface obtained by removing one surface O atom (green). The grey, light pink and red balls represent the Ni, Mo and O atoms, respectively.

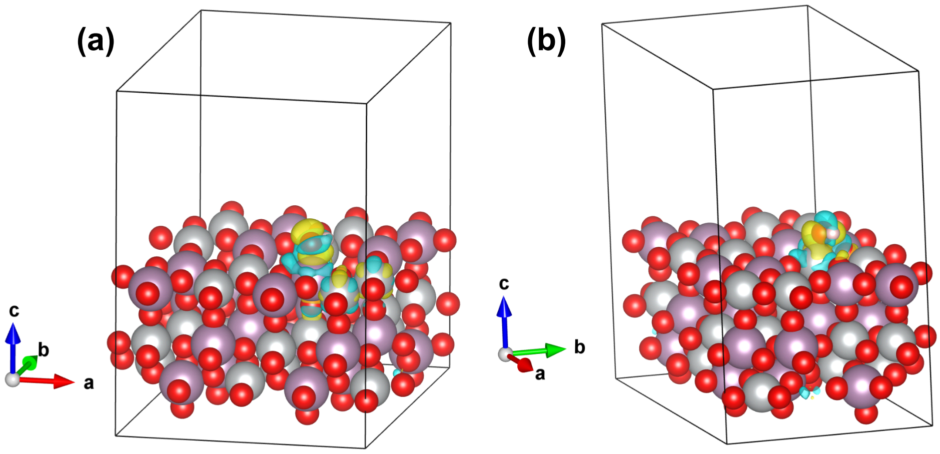

Figure S7. Charge density difference contours of after adsorption of one HO molecule on (**a**) Mo- and (**b**) Ni-side of $NiMoO_4$ (110) surface, respectively. The yellow and cyan colors denote the electron gain and loss, respectively. The isosurface level is 0.001 e bohr^{-3}.

Table S1. Brief comparison of electrochemical performance of current work with recently relevant literature.

Electrode materials	Specific capacity (F g^{-1})	Cycling stability (Cycles)	Ref.
**$NiMoO_4$/AC**	**1720 F g^{-1} at 0.8 A g^{-1}**	**84.5% (6000)**	**This work**
groove-like $NiMoO_4$ hollow nanorods	1102.2 F g^{-1} at 1 A g^{-1}	90% (1000)	[5]
Co_3O_4@$NiMoO_4$	1476 F g^{-1} at 1 A g^{-1}	96% (2000)	[6]
$NiMoO_4$/$CoMoO_4$	1445 F g^{-1} at 1 A g^{-1}	78.8% (3000)	[7]
CNTs/C/$NiMoO_4$	1037 F g^{-1} at 1 A g^{-1}	NA	[8]
CNF/$NiMoO_4$	1675 F g^{-1} at 1 A g^{-1}	83.6% (3000)	[9]
NMO@CC	970 F g^{-1} at 2.5 A g^{-1}	91.34% (5000)	[10]
NG/$NiMoO_4$	62.5 F g^{-1} at 0.5 A g^{-1}	94.2% (5000)	[11]
$NiMoO_4$/rGO	1400 F g^{-1} at 1 A g^{-1}	91% (2000)	[12]
$Ni_{0.85}Co_{0.15}MoO_4$	1301 F g^{-1} at 1 A g^{-1}	86% (3000)	[13]
$NiMoO_4$@Ni_9S_8/MoS_2	488.9 F g^{-1} at 1 A g^{-1}	81.0% (10000)	[14]
rGO/$NiMoO_4$	680 F g^{-1} at 3 A g^{-1}	68% (4000)	[15]
NMO-NS	1221.2 F g^{-1} at 1 A g^{-1}	89.2% (10000)	[16]
NiO@$NiMoO_4$@PPy	1645.1 F g^{-1} at 1 A g^{-1}	77.1% (30000)	[17]

NA: not available.

References

1. Perdew; Burke; Ernzerhof, Generalized Gradient Approximation Made Simple. *Phys. Rev. Lett.* 1996, 77, 3865-3868.
2. Kresse, G.; Joubert, D., From ultrasoft pseudopotentials to the projector augmented-wave method. *Phys. Rev. B* 1999, 59, 1758-1775.
3. Momma, K.; Izumi, F., VESTA 3 for three-dimensional visualization of crystal, volumetric and morphology data. *J. Appl. Cryst.* 2011, 44, 1272-1276.
4. Wang, V.; Xu, N.; Liu, J. C.; Tang, G.; Geng, W. T., VASPKIT: A user-friendly interface facilitating high-throughput computing and analysis using VASP code. *Comp. Phys. Comm.* 2021, 267, 108033.
5. Lin, L.; Liu, T.; Liu, J.; Sun, R.; Hao, J.; Ji, K.; Wang, Z., Facile synthesis of groove-like $NiMoO_4$ hollow nanorods for high-performance supercapacitors. *Appl. Surf. Sci.* 2016, 360, 234-239.
6. Zhao, Y.; Zhang, P.; Fu, W.; Ma, X.; Zhou, J.; Zhang, X.; Li, J.; Xie, E.; Pan, X., Understanding the role of Co_3O_4 on stability between active hierarchies and scaffolds: an insight into $NiMoO_4$ composites for supercapacitors. *Appl. Surf. Sci.* 2017, 416, 160-167.
7. Nti, F.; Anang, D. A.; Han, J. I., Facilely synthesized $NiMoO_4$/$CoMoO_4$ nanorods as electrode material for high performance supercapacitor. *J. Alloy. Compd.* 2018, 742, 342-350.
8. Xuan, H.; Xu, Y.; Zhang, Y.; Li, H.; Han, P.; Du, Y., One-step combustion synthesis of porous CNTs/C/$NiMoO_4$ composites for high-performance asymmetric supercapacitors. *J. Alloy. Compd.* 2018, 745, 135-146.
9. Huang, Y.; Cui, F.; Zhao, Y.; Lian, J.; Bao, J.; Li, H., Controlled growth of ultrathin $NiMoO_4$ nanosheets on carbon nanofiber membrane as advanced electrodes for asymmetric supercapacitors. *J. Alloy. Compd.* 2018, 753, 176-185.
10. Hussain, S.; Javed, M. S.; Asim, S.; Shaheen, A.; Khan, A. J.; Abbas, Y.; Ullah, N.; Iqbal, A.; Wang, M.; Qiao, G., Novel gravel-like $NiMoO_4$ nanoparticles on carbon cloth for outstanding supercapacitor applications. *Ceram. Int.* 2020, 46, 6406-6412.
11. Feng, X.; Ning, J.; Wang, D.; Zhang, J.; Xia, M.; Wang, Y.; Hao, Y., Heterostructure arrays of $NiMoO_4$ nanoflakes on N-doping of graphene for high-performance asymmetric supercapacitors. *J. Alloy. Compd.* 2020, 816, 152625.
12. Murugan, E.; Govindaraju, S.; Santhoshkumar, S., Hydrothermal synthesis, characterization and electrochemical behavior of $NiMoO_4$ nanoflower and $NiMoO_4$/rGO nanocomposite for high-performance supercapacitors. *Electrochim. Acta* 2021, 392, 138973.
13. Zhong, Y.; Liu, T.; Zhang, A.; Cui, L.; Liu, X.; Zheng, R.; Liu, J., Controllable synthesis of $Ni_{1-x}Co_xMoO_4$ with tunable morphologies for high-performance asymmetric supercapacitors. *J. Alloy. Compd.* 2021, 850, 156734.
14. Chen, L.; Deng, W.; Chen, Z.; Wang, X., Hetero-architectured core–shell $NiMoO_4$@Ni_9S_8/MoS_2 nanorods enabling high-performance supercapacitors. *J. Mater. Res.* 2022, 37, 284-293.
15. Muthu, D.; Vargheese, S.; Haldorai, Y.; Kumar, R. T. R., $NiMoO_4$/reduced graphene oxide composite as an electrode material for hybrid supercapacitor. *Mater. Sci. Semicond. Proc.* 2021, 135, 106078.
16. Peng, S.; Li, L.; Wu, H. B.; Madhavi, S.; Lou, X. W., Controlled growth of $NiMoO_4$ nanosheet and nanorod arrays on various conductive substrates as advanced electrodes for asymmetric supercapacitors. *Adv. Energy Mater.* 2015, 5, 1401172.
17. Yi, T.-F.; Qiu, L.-Y.; Mei, J.; Qi, S.-Y.; Cui, P.; Luo, S.; Zhu, Y.-R.; Xie, Y.; He, Y.-B., Porous spherical NiO@ $NiMoO_4$@PPy nanoarchitectures as advanced electrochemical pseudocapacitor materials. *Sci. Bull.* 2020, 65, 546-556.
